# SVM-Based Spectral Analysis for Heart Rate from Multi-Channel WPPG Sensor Signals

**DOI:** 10.3390/s17030506

**Published:** 2017-03-04

**Authors:** Jiping Xiong, Lisang Cai, Fei Wang, Xiaowei He

**Affiliations:** College of Mathematics, Physics and Information Engineering, Zhejiang Normal University, Jinhua 321004, China; 15268637225@139.com (L.C.); 18267909521@163.com (F.W.); jhhxw@zjnu.edu.cn (X.H.)

**Keywords:** adaptive filter, compressive sensing, heart rate estimation, wrist-type photoplethysmography (WPPG), principle component analysis (PCA), support vector machine (SVM)

## Abstract

Although wrist-type photoplethysmographic (hereafter referred to as WPPG) sensor signals can measure heart rate quite conveniently, the subjects’ hand movements can cause strong motion artifacts, and then the motion artifacts will heavily contaminate WPPG signals. Hence, it is challenging for us to accurately estimate heart rate from WPPG signals during intense physical activities. The WWPG method has attracted more attention thanks to the popularity of wrist-worn wearable devices. In this paper, a mixed approach called Mix-SVM is proposed, it can use multi-channel WPPG sensor signals and simultaneous acceleration signals to measurement heart rate. Firstly, we combine the principle component analysis and adaptive filter to remove a part of the motion artifacts. Due to the strong relativity between motion artifacts and acceleration signals, the further denoising problem is regarded as a sparse signals reconstruction problem. Then, we use a spectrum subtraction method to eliminate motion artifacts effectively. Finally, the spectral peak corresponding to heart rate is sought by an SVM-based spectral analysis method. Through the public PPG database in the 2015 IEEE Signal Processing Cup, we acquire the experimental results, i.e., the average absolute error was 1.01 beat per minute, and the Pearson correlation was 0.9972. These results also confirm that the proposed Mix-SVM approach has potential for multi-channel WPPG-based heart rate estimation in the presence of intense physical exercise.

## 1. Introduction

Heart rate estimation can provide useful information for users who are engaged in rehabilitation or in physical exercise and anyone who wants to routinely keep track of their cardiac status. Traditional heart rate estimation mainly relies on electrocardiogram (ECG), but ECG requires the presence of ground and reference sensors that must be attached to the body. That is to say, its application area was limited because of the high hardware complexity and low user comfort ability. Photoplethysmography (PPG) [1,2] was widely used for measuring blood volume changes in tissue due to its non-invasive nature and low cost. Unfortunately, the quality of PPG sensor signals can be easily affected by motion artifacts during intensive physical exercise. Therefore, the motion artifacts must be removed to measure heart rate accurately [3,4,5].

In order to improve the quality of PPG sensor signals with motion artifacts, researchers have conducted several explorations to address the above problem, including adaptive noise cancellation (ANC) [6,7,8], singular value decomposition [9], time and period domain analysis [10], wavelet-based method [11,12], blind source separation (BBS) [13], spectrum subtraction [14], empirical mode decomposition (EMD) [15,16], Kalman filtering [17], and Wigner-Ville distribution [18], to name a few. However, the above methods can work well under mild-movement conditions only where motion artifacts are not strong (walking, jogging, finger movements).

Lately, Lopez-Silva et al. [19] proposed a heuristic algorithm, its first step was the linear filtering of WPPG signal via band-pass Bessel filtering to get the pulsating component and to suppress high-frequency noise and ripples. Then a frequency-domain analysis was performed by applying the FFT. Finally, a peak search algorithm was applied to the stream of spectra to select peaks that are above the noise floor. Lai [20] proposed a novel heart rate monitoring method for heavy exercise. There were four sets of algorithms as the building blocks of the proposed method: preprocessing, signal representation using short-time Fourier transform, detection and ranking of potential cardiac and motion components, and robust heart rate estimation. Meanwhile, a new method called TROIKA was presented by Zhang et al. [21,22]. The method can estimate heart rate from WPPG signal for scenarios where motion artifacts are strong. It was based on single measurement vector (SMV) model and spectral peak tracking. Firstly, the algorithm utilized signal decomposition and SVM model to remove the strong motion artifacts in the WPPG signal. Then the spectral peaks corresponding to heart rate can be determined by spectral peak tracking. As a sequel to this study, Zhang proposed the variant of TROIKA framework, which was named JOSS [23]. It was based on a multiple measurement vector (MMV) model, spectrum subtraction, and spectral peak tracking. It further improves the previous results by jointly exploiting the spectral structures of WPPG signal and the accelerometer signals. Motivated by the aforementioned model, Xiong et al. [24] presented an approach, denoted by MC-SMD. In MC-SMD, a spectral matrix is constructed by using multi-channel WPPG sensor signals and simultaneous acceleration signals. The spectral matrix is divided into two matrixes—i.e., motion artifacts matrix and real PPG sensor signals matrix. At last, heart rate estimates are output by the proposed spectral peak tracking method. Tautan et al. [25] proposed a heart rate measurement algorithm using duel color PPG. There were six signals in the algorithm: $PPG_{i}$, the five reference signals $A_{x,y,z,M}$ and $PPG_{ref}$. Then the algorithm comprised three stages: preprocessing, reference and threshold selection, and wavelet decomposition and reconstruction. However, the main drawbacks of the above means are that the spectral peak tracking procedure involves many user-tuning parameters. Thus, their generalization performance becomes poor, and the computational load is heavy.

In this paper, we propose an accurate and efficient strategy, named Mix-SVM, which tracks subjects’ heart rate based on a mixed method composed of adaptive filter, sparse signal reconstruction model, spectrum subtraction, and SVM-based spectral analysis. Firstly, we generate a reference motion artifacts signal component by dealing with the acceleration signals by principal component analysis (PCA) prior to adaptive filtering. Then, we use adaptive filtering to reduce part of the motion artifacts. Next, we construct a sparse signal reconstruction model and utilize spectrum subtraction to further reduce motion artifacts. Finally, we use an SVM-based spectral analysis approach to solve the spectral peak tracking problem.

In this article, the materials and process of our Mix-SVM method is outlined in Section 2. The evaluation results of the simulation experiment are given in Section 3. Conclusions are drawn in the last section.

## 2. Materials and Methods

### 2.1. Data Description

The 12 data sets are introduced as part of the 2015 IEEE Signal Process Cup [26] and used by various researchers. These data all are recorded from 12 healthy Asian males ages 18 to 35. The each dataset simultaneously recorded two-channel WPPG signals, three-channel acceleration signals and single-channel ECG signal. Two pulse oximeters with green LEDs (wavelength: 515 nm) and a tri-accelerometer (the instrument can measure the space acceleration and fully reflect the kinetic property of objects) were used to gather the WPPG signals and acceleration signals, respectively. Both the pulse oximeters and the accelerometer were embedded in a wristband, which was comfortably worn. Meanwhile, the wet ECG sensors were used for acquiring the ground-truth of heart rate on the chest.

The data sets were gathered while 12 healthy male participants walked or ran on a treadmill. They first start from rest to high speed, i.e., 1–2 km/h for 30 s, 6–8 km/h for 60 s, 12–15 km/h for another 60 s. Next, the same cycle is repeated, i.e., starting at speed of 6–8 km/h for 60 s, followed by 12–15 km/h for 60 s. Finally, these subjects slow down to 1–2 km/h for 30 s.

### 2.2. Methodology

**Figure 1:**
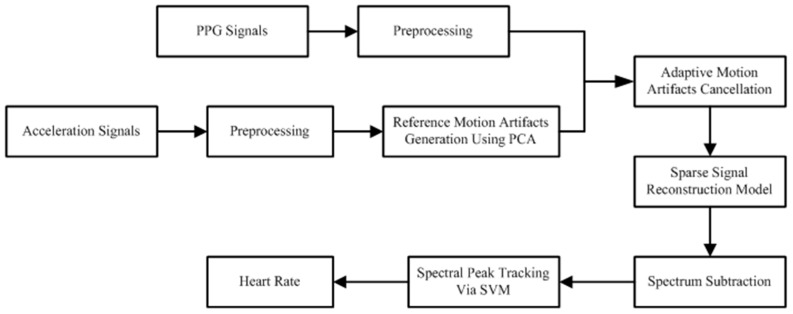
Flowchart of the developed system.

As mentioned earlier, our proposed method is comprised of five main stages: preprocessing, initial motion artifact reduction, sparse signal reconstruction model, spectrum subtraction, and SVM-based spectral analysis. Figure 1 shows the flowchart of the developed system. The blocks used in the proposed method are introduced in the following subsection.

#### 2.2.1. Preprocessing

Heart rate can be estimated in a time window when the simultaneous multi-channel WPPG sensor signals and acceleration signals are slide in the time window. In our experiments, the time window was set to 8 s and two successive time windows overlap by 6 s.

At the beginning of the preprocessing, the frequency of the multi-channel WPPG sensor signals and the acceleration signals are 125 Hz, their sampling frequency becomes 25 Hz by downsampling. The above process can facilitate the subsequent activity.

Generally, health adults’ heart rates vary from 0.5 Hz to 3 Hz. Therefore, the multi-channel WPPG sensor signals and the acceleration signals all are filtered with the second order Butterworth band-pass filter (0.4 Hz–4 Hz). Then, a majority of noise is removed outside the frequency band because of the above filtering procedure.

#### 2.2.2. Initial Motion Artifacts Reduction

In this subsection, prior to further denoising processing, we apply an adaptive filter to remove a part of motion artifacts in multi-channel WPPG sensor signals. Although adaptive noise cancellation was already utilized by [27,28] to remove motion artifacts, the reference motion artifact signals were extracted from the PPG signal itself. This approach may work sufficiently for low motion artifact scenarios, but for intensive physical exercise, the reference motion artifacts signal components needed for the adaptive filter are extracted from the simultaneous acceleration signals by principle component analysis (PCA) [29]. The two substeps will be explained in the following details:

*Reference Motion Artifacts Generation Using PCA*: The simultaneous acceleration signals along the three axes include footprints of the motion artifact. However, the acceleration data are convoluted noisy signals composed of different periodic components themselves. If we utilize them directly as the motion artifacts reference, this will strongly hamper convergence of the adaptive filter and degrade the overall performance. Hence, we use PCA to analyze the acceleration signals, and then choose the first principal component which contains the most “information” as the reference motion artifact signal components. In this technique, each acceleration signal goes through the following steps: first, the acceleration signals are standardized; second, we calculate the correlation coefficient matrix; third, we use the Jacobian method to compute the eigenvalues of the correlation coefficient matrix; fourth, according to contribution rate, we select the important principal components; finally, we calculate the scores of these principal components and acquire the reference motion artifact signal components.

*Adaptive Filter for Motion Artifacts:* During this procedure, all the reference motion artifact components are used in the adaptive least mean square (LMS) filter. We continually update the weight of the filter according to the criterion of mean square error minimization. Then a part of the motion artifacts from multi-channel WPPG sensor signals can be eliminated. Suppose the observed WPPG signal

(1) $$y(l)=y_{0}(l)+m(l).$$

where $y_{0}(l)$ is the cleansed WPPG signal and m(l) is the motion artifact signals.

Let the difference value e(l) and the weight w(l) update be expressed as follows

(2) $$\begin{array}{l} e(l)=y_{0}(l)+m(l)-m\prime (l);\\ m\prime (L)=w(l)\times a(l);\\ w(l+1)=w(l)-\mu \times e(l)\times a(l). \end{array}$$

where m′(l) denotes the estimated motion artifact, a(l) is the reference motion artifact signal components described above, and μ is a parameter which determines the stability and convergence of iteration.

#### 2.2.3. Sparse Signal Reconstruction Model

At first, the sparse spectrum of a raw PPG signal can be estimated by SVM model [21,22]. The expression of the basic model is as follows

(3) y=Φx+v.

where $y\in R^{M\times 1}$ is an observed signal, $x\in C^{N\times 1}$ is assumed to be sparse, and it is also an unknown vector, $\Phi_{m,n}=e^{j\frac{2\pi }{N}mn}\in C^{M\times N}(M<N)$ is the redundant discrete Fourier transform (DFT) basis, and $v\in R^{M\times 1}$ is the modeling errors.

Based on SVM model, the *i*-th spectrum coefficient of the PPG signal is estimated $s_{i}$

(4) $$s_{i}={\left|{\overset{⌢}{x}}_{i}\right|}^{2},\begin{array}{c} i=1,2,\ldots ,N \end{array}.$$

where ${\overset{⌢}{x}}_{i}$ is the *i*-th element of $\overset{⌢}{x}$.

Then, the MMV model [24] can use the multiple measurement vectors to estimate a solution jointly. So the objective function of the model is as follows

(5) Y=ΦX+V.

where $Y\in R^{M\times H}$ is the measurement matrix, $\Phi_{m,n}$ also is the redundant DFT basis, $X\in C^{N\times H}$ is the sparse matrix, and $V\in R^{M\times H}$ represents model errors.

Through the above analysis, the paper proposes a sparse signal reconstruction model for further denoising, which is based on compressive sensing theory. Owing to the strong relativity between motion artifacts in multi-channel WPPG sensor signals and simultaneous acceleration signals, we can extract the sparse characteristics of rows of the spectral matrix. Hereby, we can write the procedure, i.e.,

(6) $$\begin{array}{l} \underset{X}{\min}\frac{1}{2}{\left\|Y-\Phi X\right\|}_{F}^{2}+\tau {\left\|X\right\|}_{1.2};\\ \begin{array}{c} \;s.t:Y=\Phi X+V \end{array}. \end{array}$$

where ${\left\|X\right\|}_{1,2}={\sum_{i=1}^{N}\left(\sum_{j=1}^{R}{x_{i,j}}^{2}\right)}^{\frac{1}{2}}$ constrains the row sparse of the spectral matrix. $x_{i,j}$ denotes the (*i*, *j*)-th entry of X. τ is a weight.

In addition, multi-channel WPPG sensor signals are contained in the measurement matrix Y. The multi-channel WPPG sensor signals have removed a part of motion artifacts via adaptive filter. In the simulation experiments, we merely adopt two WPPG sensor channel signals (namely, WPPG1, WPPG2, respectively).

It is well known that this model can utilize many proposed algorithms to attain a solution [30,31]. However, the adjacent columns of the matrix Φ always are highly coherent, so not every algorithm is suitable. In this paper, the solution of this model is estimated by utilizing the Regularized M-FOCUSS algorithm [32], the mathematical expression of the algorithm is described as follows

(7) $$\begin{array}{l} W_{k+1}=diag(c_{k}{\left[i\right]}^{1-\frac{p}{2}});\\ Q_{k+1}=A_{k+1}^{+}B;\\ X_{k+1}=W_{k+1}Q_{k+1}. \end{array}$$

where $c_{k}[i]={\left(\sum {\left(x_{k}^{\left(l\right)}[i]\right)}^{2}\right)}^{\frac{1}{2}}$ and $A_{k+1}=AW_{k+1}$.

The M-FOCUSS algorithm is an extension of the FOCUSS class of algorithms. It can quickly converge when the large coefficients are observed [33]. Hence, even if a high correlation matrix Φ exists, the algorithm still presents fast and reliable performance.

#### 2.2.4. Spectrum Subtraction

Thanks to the spectral peak positions of motion artifacts in multi-channel WPPG spectra and the spectral peak positions of the acceleration spectra alignment, we can use the acceleration spectra to subtract the motion artifacts from multi-channel WPPG spectra. The operation mode of spectrum subtraction is similar to the proposed process in [23]. Here, given two WPPG spectra and three acceleration spectra.

Step 1: We need to seek the maximum value $Acc_{l}$ of spectral coefficients in acceleration spectra for each frequency bin $f_{l}(l=1,2,\ldots ,N)$.
Step 2: At each frequency bin $f_{l}(l=1,2,\ldots ,N)$, the value of the spectral coefficients are subtracted $Acc_{i}$ in two WPPG spectra. Within $0\leq f_{l}\leq 199$, the maximum values of all coefficients are denoted $p_{\max1}$, $p_{\max2}$ in two WPPG spectra, respectively.
Step 3: Within $0\leq f_{l}\leq 199$, we set to zero all spectral coefficients with values less than $p_{\max1}/5$ in WPPG1 spectra, and all spectral coefficients with values less than $p_{\max2}/5$ are also set to zero in WPPG2 spectra. Finally, we can yield two cleansed WPPG spectra.

Because the heart rate values are less than 180 beat per minute (BPM) in most conditions (including intense exercises), and the maximum recorded heart rate value is 230 BPM, we just analyze the WPPG spectra within $0\leq f_{l}\leq 199$. Moreover, WPPG spectra and acceleration spectra should be normalized to have the same energy prior to spectrum subtraction method.

#### 2.2.5. SVM-Based Spectral Analysis

After the aforementioned stages, motion artifacts are mainly removed in multi-channel WPPG sensor signals. The next step is finding the related spectral peak of heart rate.

Here, we put forward a spectral analysis approach based on the Support Vector Machine (SVM) approach [32]. In this approach, the spectral peak tracking is regarded as a two-category classification task. In addition, we also fully consider the statistical properties of the multi-channel WPPG sensor signals. Then, the procedure of spectral analysis has two steps: spectral peak discovery and spectral peak selection.

In the different WPPG spectra, the possible peaks are found by spectral peak discovery. That is to say, an adaptive threshold κ automatically is set to

(8) κ=ξ⋅max{|x|}.

where x denotes the spectrum of each denoised WPPG channel, ξ is a parameter, and max{⋅} is a mathematical operation, it can extract the maximum value.

Then, a candidate spectral peak set is formed, i.e., when the frequencies of spectral peaks with coefficients larger than the threshold κ, it is included the previous proposed set.

The goal of spectral peak selection is to determine the best reliable spectral peak corresponding to heart rate from the candidate spectral peak set. To the best of our knowledge, there is only one true spectral peak in each time window. There are many different features between the true spectral peak and false spectral peaks. Researchers have investigated the statistical properties of the true peaks, so these effective features showed that [34]

(1) About 75% of spectral peaks that have the largest coefficient in their corresponding time windows are true spectral peaks.
(2) About 84% of spectral peaks that have the shortest distance from their previous true spectral peak are true spectral peaks.
(3) About 96% of true spectral peaks have the largest coefficient and shortest distance.

In conclusion, we choose two traits to quantify differences among the candidate spectral peaks. The traits are the peak coefficient ratio and peak-to-peak distance, respectively. In the current time window, the candidate spectral peak set has *L* candidate spectral peaks. While the *l*-th candidate spectral peak’s coefficient ratio is defined as

(9) $$C_{l}=\left|\frac{coe_{l}}{coe_{\max}}\right|,l=1,\ldots ,L$$

where $coe_{l}$ is the peak coefficient of the *l*-th candidate spectral peak and $coe_{\max}$ is the maximum peak coefficient in the candidate spectral peak set.

Also, the peak-to-peak distance of the *l*-th candidate spectral peak is defined as

(10) $$S_{l}=\left|f_{l}-f_{prev}\right|,l=1,\ldots ,L$$

where $f_{l}$ is the frequency of the *l*-th candidate spectral peak and $f_{prev}$ is the true estimated peak in the previous time window.

In the view of the accuracy and robustness to noise of SVM, it is often used for classification and regression problems. Meanwhile, SVM has high generalization performance due to the special properties of the decision surface. Therefore, we adopt SVM to classify true spectral peaks from false ones. In the training phase, we firstly extract the two features described above from all candidate spectral peaks. Then the true spectral peaks are labeled as “1” and the false spectral peaks are labeled as “0”. Next, the SVM trains itself with the labeled features and finds the support vectors among the features. Finally, a decision boundary is determined which is based on the support vectors.

In test phase, we first collect two features as before, and form feature vectors. Next, a trained SVM classifier is developed to examine whether they are true spectral peaks. The specific choice rules are as follows

(1) If only one spectral peak is true, and then we select the spectral peak corresponding to frequency, denoted as $f_{HR}$.
(2) If the classifier output more than one true spectral peak, we select the spectral peak of the closest to $f_{pre}$, its frequency is denoted as $f_{HR}$.
(3) If there is no true spectral peak, we consider that SVM classifier cannot seek out reliable spectral peaks because of serious motion artifacts in the current time window. Hence, a prediction mechanism is proposed for solving the problem. The mechanism can be expressed as follows
  (11) $$f_{HR}=\left\{ \begin{array}{ll} f_{pre}+0.02 & if\;h>0;\\ f_{pre}-0.02 & if\;h<0;\\ f_{pre} & if\;h=0. \end{array}\right.$$

where $h=predict-predict_{pre}$, predict is the current predicted frequency, and $predict_{pre}$ is the previous predicted frequency. Here, predict and $predict_{pre}$ are computed by the Smoother algorithm [35]. It is operated on the frequencies of the 10 closest previously estimated heart rate values. Once $f_{HR}$ is determined, the current estimated heart rate $BPM_{est}$ can acquire via

(12) $$BPM_{est}=f_{HR}\times 60.$$

## 3. Results

### 3.1. Parameter Settings

We choose an adaptive LMS filter of order 25 to reduce a part of motion artifacts with an optimized μ=0.005. In a sparse signal reconstruction model, the weighting parameter τ=1. Then, we set the regularization parameter $\lambda =10^{-10}$ in the Regularized M-FOCUSS algorithm [33], and the spectrum grid number N=1024. For the SVM-based spectral peak selection, the parameter in Equation (8) to ξ=0.7, and the smoother parameter of the Smoother algorithm [36] was set to 20.

Note that the SVM classifier is trained by using five training data and five test data from the public datasets [26]. Meanwhile, we choose all training data to appraise the performance of Mix-SVM.

### 3.2. Data Analysis and Statistics

To measure the performance of Mix-SVM, we adopt the average absolute error, average absolute error percentage, Bland-Altman plot, and Pearson correlation to analyze the relationship between estimates and ground-truth values. Let the ground-truth values and the estimates be $BPM_{true}(l)$, $BPM_{est}(l)$ in the *l*-th time window, respectively, and the total number of time window be W. Then, the average absolute error (AAE) was calculated as

(13) $$AAE=\frac{1}{W}\sum_{l=1}^{W}\left|BPM_{est}\left(l\right)-BPM_{true}\left(l\right)\right|.$$

In the same way, the average absolute error percentage (AAEP) was computed as

(14) $$AAEP=\frac{1}{W}\sum_{l=1}^{W}\frac{\left|BPM_{est}\left(l\right)-BPM_{true}\left(l\right)\right|}{BPM_{true}\left(l\right)}.$$

The agreement between ground truth values and estimates was directly reflected by Bland-Altman plot [35]. After analyzing the limit of agreement is expressed LOA=[u−1.96σ,u+1.96σ], and u, σ denote the average difference and the standard deviation respectively. At last, Pearson correlation can examine whether ground-truth values and estimates locate in a line.

As we all know, the classification accuracy is the most important indicator for evaluating the performance of the classifier. Thus, we used the effective 10-fold cross-validation method in our experiment. In this process, the public datasets were divided into 10 subsets. Every subset was considered as the validation set, the rest of the subsets as training set, and then we obtained 10 classification models. Finally, the mean of the classification accuracy of the 10 models can be used as a measurement indicator.

### 3.3. Results Analysis

For direct comparison, Table 1 and Table 2 give AAE and AAEP of Mix-SVM, TROIKA [21,22], JOSS [23], and MC-SMD [24] method on all datasets, respectively. The results show that the performance of Mix-SVM was entirely superior to TROIKA over the 12 datasets. We can also observe that Mix-SVM had better nature than JOSS and MC-SMD on most of datasets. Furthermore, we compute the averages of AAE and AAEP for aforementioned four algorithms across the 12 datasets. Where Mix-SVM were 1.01 BPM and 0.72%. In TROIKA framework, AAE = 2.42 BPM and AAEP = 1.82%. In JOSS, AAE = 1.28 BPM, and AAEP = 1.01%. In MC-SMD, AAE = 1.11 BPM and AAEP = 0.80%.

**Figure 2:**
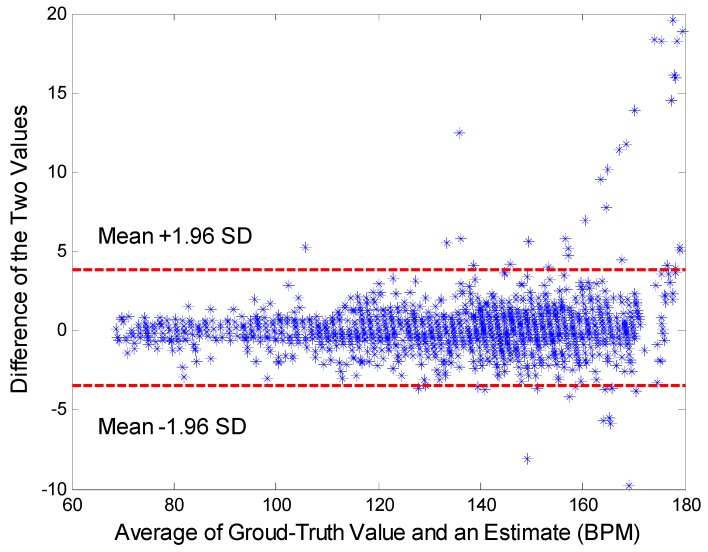
Bland-Altman plot.

**Figure 3:**
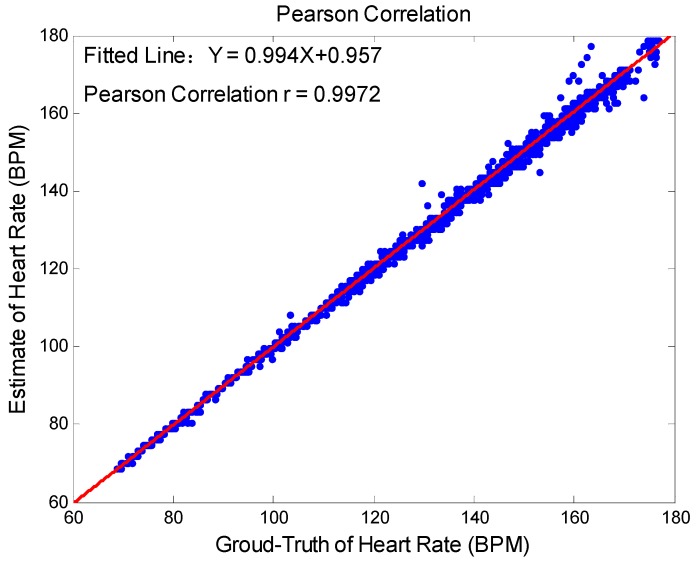
Scatter plot.

Among 12 datasets, Figure 2 depicts the Bland-Altman plot, where LOA=[−3.46,3.83] BPM. In order to observe the relationship between the ground-truth heart rate values and the associated estimates, we give the scatter plot (Figure 3). Then Y=0.994X+0.957 was the fitted line, where *X* denotes the ground truth values of heart rate, and *Y* the estimated heart rate. The Pearson coefficient was 0.9972.

**Figure 4:**
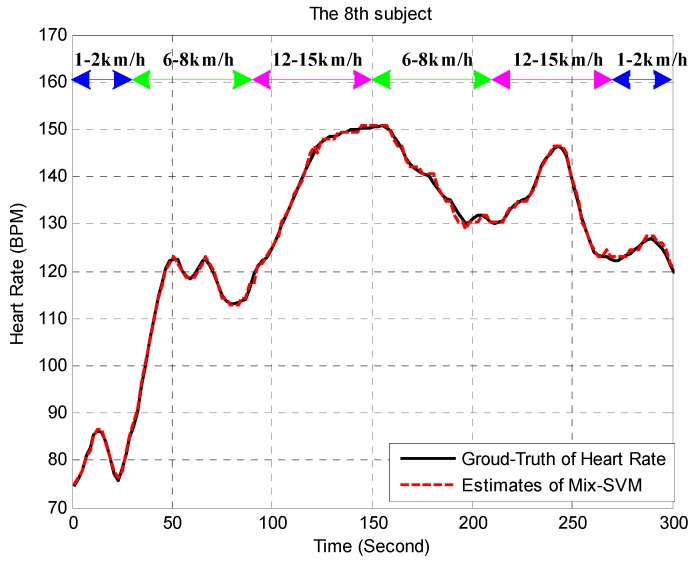
Estimation result on subject 8.

To get a closer look to the ability of Mix-SVM, Figure 4 presents an example of estimate of Mix-SVM on subject 8 (randomly chosen). We can find that the estimates were quite close to the ground truth values as we expected.

**Figure 5:**
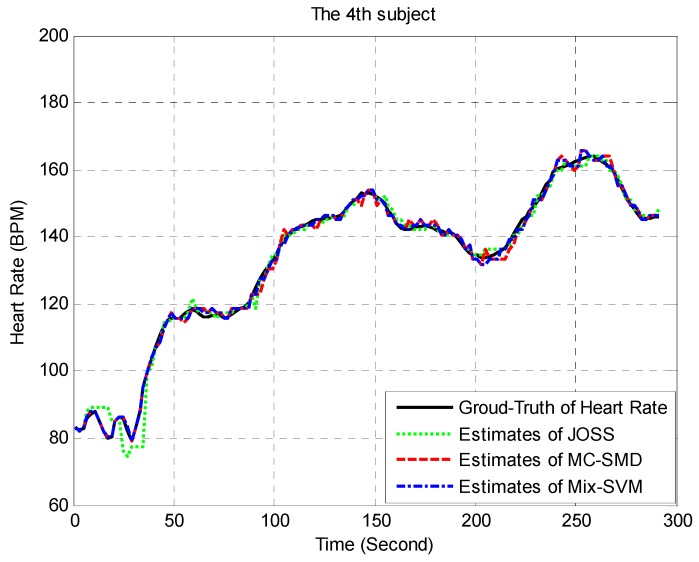
Estimation results on subject 4 for JOSS, MC-SMD, and Mix-SVM.

To show the superiority of our proposed algorithm, the estimated heart rate traces of Mix-SVM, JOSS, and MC-SMD on subject 4 (randomly chosen) is shown in Figure 5. Mix-SVM had the best performance among three methods, its estimated heart rate trace almost overlaps with the ground-truth of heart rate trace, but JOSS and MC-SMD sometimes got errors. For example, the estimates of JOSS have gravely deviated from the ground-truth of heart rate from 0 to 50 s. From 110 to 115 s, 140 to 145 s, 210 to 225 s and 265 to 270 s, the estimates of MC-SMD do not overlap with the ground-truth value. But the estimates of Mix-SVM were nearly equal to the ground-truth of heart rate. Due to the performance of our proposed method being entirely superior to TROIKA on each subject, we ignore the estimated heart rate traces of TROIKA in Figure 5.

## 4. Conclusions

A novel mixed approach termed Mix-SVM was developed for estimation of heart rate from multi-channel WPPG signals with various types of motion artifacts. In this approach, we used the fast denoising algorithm and reconstruction algorithm to deal with serious motion artifacts caused by users’ fast activities. Then we utilized the SVM-based method to analyze spectra. Through the simulation experiment on the 12 datasets, the results verified that the accuracy and efficacy of Mix-SVM, and the estimates of Mix-SVM were close to the ground-truth of heart rate. Furthermore, SVM can seek the optimal solution according to finite sample information. The theoretical basis of SVM determines that the final solution is the global optimal solution rather than the local minimum value. The above features ensure the good generalization ability of SVM for unknown samples. Hence, Mix-SVM may greatly improve its own generalization ability.

## Figures and Tables

**Table 1:** The AAE on the 12 datasets. The unit is BPM.

Methods	S1	S2	S3	S4	S5	S6	S7	S8	S9	S10	S11	S12	Average
Literature [22]	2.87	2.75	1.91	2.25	1.69	3.16	1.72	1.83	1.58	4.00	1.96	3.33	2.42
Literature [23]	1.33	1.75	1.47	1.48	**0.69**	1.32	**0.71**	0.56	0.49	3.81	**0.78**	1.04	1.28
Literature [24]	1.16	1.07	0.80	1.13	0.98	1.29	0.88	0.81	0.55	3.18	0.79	**0.72**	1.11
Mix-SVM	**1.08**	**1.00**	**0.69**	**0.86**	0.80	**1.24**	0.90	**0.52**	**0.48**	**2.95**	0.80	0.75	**1.01**

**Table 2:** The AAEP on the 12 datasets. The unit is %.

Methods	S1	S2	S3	S4	S5	S6	S7	S8	S9	S10	S11	S12	Average
Literature [22]	2.18	2.37	1.50	2.00	1.22	2.51	1.27	1.47	1.28	2.49	1.29	2.30	1.82
Literature [23]	1.19	1.66	1.27	1.41	**0.51**	1.09	**0.54**	0.47	0.41	2.43	**0.51**	0.81	1.01
Literature [24]	0.91	0.87	0.62	0.84	0.68	0.96	0.65	0.64	0.43	1.95	**0.51**	**0.53**	0.80
Mix-SVM	**0.86**	**0.81**	**0.53**	**0.65**	0.55	**0.92**	0.66	**0.42**	**0.40**	**1.79**	0.52	0.55	**0.72**
